# Central precocious puberty in a girl with LEGIUS syndrome: an accidental association?

**DOI:** 10.1186/s13052-021-01004-9

**Published:** 2021-03-04

**Authors:** Valentina Orlandi, Paolo Cavarzere, Laura Palma, Rossella Gaudino, Franco Antoniazzi

**Affiliations:** 1 Pediatric Division, Department of Pediatrics, University Hospital of Verona, Piazzale Stefani 1, 37126 Verona, Italy; 2 Pediatric Clinic, Department Surgical Sciences, Dentistry, Gynecology and Pediatrics, University of Verona, Verona, Italy; 3 Regional Center for the diagnosis and treatment of children and adolescents rare skeletal disorders. Pediatric Clinic, Department of Surgical Sciences, Dentistry, Gynecology and Pediatrics, University of Verona, Verona, Italy

**Keywords:** Legius syndrome, Precocious puberty, Rasophaties, Neurofibromatosis type 1

## Abstract

**Background:**

Central precocious puberty is a condition characterized by precocious activation of the hypothalamic-pituitary-gonadal axis. It may be idiopathic or secondary to organic causes, including syndromes such as Neurofibromatosis type 1 (NF1).

**Case presentation:**

We presented a girl of 6 years and 10 months with almost 11 café-au-lait skin macules, without other clinical or radiological signs typical of NF1, and with a central precocious puberty. Genetic analysis evidenced the new variant NM-152594.2:c.304delAp. (Thr102Argfs*19) in *SPRED1* gene, which allowed to diagnose Legius syndrome.

**Conclusions:**

We report for the first time a case of central precocious puberty in a girl with Legius syndrome. The presence of central precocious puberty in a child with characteristic café-au-lait macules should suggest pediatricians to perform genetic analysis in order to reach a definitive diagnosis. Further studies on timing of puberty in patients with RASopathies are needed to better elucidate if this clinical association is casual or secondary to their clinical condition.

## Background

Precocious puberty (PP) is a condition characterized by the appearance of pubertal signs before age 8 for girls and age 9 for boys and is 8 times more frequent in females than in males [1–4]. Clinically, PP causes early development of secondary sexual characteristics, rapid bone maturation, increased growth velocity, behavioral changes, inappropriate physical aspect for the chronological age, and reduction in adult height [4].

It is possible to distinguish a central precocious puberty (CPP), GnRH-dependent, from a peripheral precocious puberty (PPP), GnRH-independent. CPP is due to a precocious activation of the hypothalamic-pituitary-gonadal axis. Although it is frequently idiopathic, it might also be secondary to organic causes, such as central nervous system tumors, injury of the central nervous system, genetic conditions or syndromes (e.g. neurofibromatosis type 1 (NF1), Sturge-Weber syndrome, and tuberous sclerosis) [1, 5]. Organic forms of CPP usually start at a younger age than the idiopathic form, with a more rapid progression. Yet, it is important to exclude neurogenic causes, especially in boys, in which idiopathic form is rarer than in girls [2–4].

In opposition to what happens with CPP, in PPP the production of sexual steroids is independent from the hypothalamic impulse. Some conditions that lead to PPP, such as familial testotoxicosis, are typical of males, others of females (ovarian cysts), finally there are forms present in both sexes, such as congenital adrenal hyperplasia, McCune-Albright syndrome (MAS) and hypothyroidism [3, 4].

## Case presentation

A girl of 6 years and 10 months arrived to our Pediatric Endocrinology Centre for a suspected PP. Parents are not related, and no noteworthy disease was reported in her familial history, in particular, no one in the family presented disorders of the sexual development.

She was born at 39 weeks of gestational age by spontaneous delivery after an uneventful pregnancy. Birth weight was 3210 g (− 0,01 standard deviations, [SD]), birth length was 49 cm (− 0,21 SD), cranial circumference was 32,5 cm (− 1,16 SD).

At birth, the presence of many café-au-lait skin macules was detected, and this feature was reported in her father and in her paternal grandmother too. For the presence of these macules, she was submitted to dermatologic, neuropsychiatric and ophthalmologic evaluations on clinical suspicion of NF1. Neuropsychiatric evaluation did not detect any relevant neuro-behavioral problem, apart from some difficulties in attention maintenance. No dermatological or ocular signs associated with NF1 were reported. In addition, no noteworthy disease was described in her clinical past.

Four months before our evaluation, parents detected the appearance of bilateral thelarche, adult body odor and leukorrhea. At our first clinical evaluation her weight was 23.2 kg (− 0,23 SD), her height was 120.9 cm (0 SD). Her growth velocity appeared accelerated (approximately 1 cm/month; > 2 SD). Her Tanner’s stage was T2, P1–2, A2. We found at least 11 café-au-lait skin macules spread in torso, arms, neck and one in forehead, with a variable diameter from 4 mm to 17 mm. No neurofibromas or freckles were detected. Her bone age compared with Greulich & Pyle charts corresponded to an age of 8 years and 6 months.

Based on these clinical findings a stimulation test with GnRH-analogue (triptorelin) was performed. Basal concentration of LH and FSH was 1,68 U/L, and 4,2 U/L respectively. After 4 h, LH peak concentration was 35 U/L while FSH peak concentration was 29,1 U/L. Basal estradiol value was 176 pmol/L, estradiol peak was 186 pmol/L. According to these results, we confirmed a CPP. Consequently, we started a treatment with GnRH-analogue every 28 days, with good response.

To complete the diagnostic investigation a brain magnetic resonance imaging (MRI) was performed, which did not present any trace of pathological findings, in particular in the pituitary region and in the optic pathway. Moreover, no cerebral sign of NF1 was evidenced. However, in order to confirm (or disprove) the suspect of NF1 we also performed genetic analysis. To identify causative mutation on the *NF1* gene, the genomic DNA was extracted from peripheral blood leukocytes and Targeted Next Generation Sequencing (NGS) was performed. Given the normality of the *NF1* gene, we analyzed the SPRED1 gene associated with the Legius syndrome, in order to prove a possible differential diagnosis.

This analysis identified the variant NM-152594.2:c.304delAp. (Thr102Argfs*19) in *SPRED1* gene. This variant in *SPRED1* is not reported in literature, but as far as we can ascertain from the characteristics it presents, it probably has pathogenetic nature. This finding finally brought to the diagnosis of Legius syndrome.

## Discussion and conclusions

This is the first case of CPP in a patient with Legius syndrome. Initially we consider her CPP a consequence of a suspected NF1; only afterwards, when a new mutation in the *SPRED1* gene was detected, it became possible to conclude that she was affected by Legius syndrome. Nowadays, it is still unknown whether Legius syndrome can predispose to the development of CPP.

Legius syndrome is a rare genetic disease included in RASophaties, a group of conditions caused by alterations in RAS/MAPK pathway, among which the Noonan syndrome is the best known. NF1 is part of this group too [5, 6]. The first case was identified by Brems in 2007 in a patient with a similar-NF1 phenotype in which the genetic analysis showed a loss-of-function mutation in *SPRED1* gene [7]. This gene encodes Spred1, a protein member of Sprouty/Spred family that acts as a negative regulator in RAS/MAPK pathway. As a consequence of this mutation, transmission of the signal is always active [5, 6]. The inheritance of Legius syndrome is autosominal dominant. It is estimated that approximately 1–4% of people with café-au-lait skin macules has this syndrome [8, 9]. In addition to café-au-lait skin macules and axillary or inguinal freckles, lipomas, macrocephaly, learning difficulties, ADHD (Attention Deficit Hyperactivity Disorder) and delay in neurobehavioral development are described [8]. Our patient presented in fact a mild disorder in attention maintenance, which might be a misunderstood sign of her syndrome.

To date, no association between PP and Legius syndrome is known. The role of pediatrician endocrinologists is essential first in recognizing a possible genetic syndromic pathology from endocrinological symptoms and secondly in managing the endocrinological complications of known genetic syndromes. The recognition might be easy under some conditions, i.e. a possible association between CPP and NF1, but more often, the connection between endocrine features and genetic syndromes remains unfamiliar, as in our clinical case [10]. In girls with clinical signs of PP it is necessary to verify the activation of the hypothalamic-pituitary-gonadal axis in order to classify their condition as central or peripheral. Furthermore, attention must be paid to every clinical element that can help to recognize a possible associated genetic condition and to identify the etiology of PP, such as visual deficit, headache, bone pain, bone deformity or dermatologic findings. The coexistence of cutaneous manifestation and clinical signs of PP suggests the possible presence of NF1 or MAS. Whereas the former condition is associated with CPP, the latter is associated with PPP [3, 4]. Moreover, these two clinical conditions differ from each other in type, characteristics and distribution of café-au-lait skin macules [11, 12]. On the basis of these data, we promptly excluded the hypothesis of MAS. Accordingly, a strong clinical suspect of NF1 remained. In fact, café-au-lait skin macules associated with Legius syndrome are clinically indistinguishable from those associated with NF1 as far as number, pattern of distribution, or characteristics are concerned. Moreover, patients with Legius syndrome, exactly like in NF1, have axillary and/or inguinal freckling [11]. The difference between these two syndromes resides in the absence of neurofibromas, Lisch nodules, optic pathway glioma, tibial dysplasia or central nervous system tumors in cases of Legius syndrome [11, 12]. These data were confirmed in our patient through brain MRI and ophthalmology evaluation, nevertheless it was only the genetic analysis that allowed to achieve the definitive diagnosis of Legius syndrome. Whereas CPP in patients with NF1 was considered for years a complication related to optic pathway gliomas, due to the involvement of the hypothalamic and sellar region [13], recent studies have described some cases of children with NF1, CPP and no cerebral lesions [14]. Therefore, in presence of a girl with café-au-lait macules and CPP, clinicians should take into consideration not only NF1 but also Legius syndrome if not all NF1 diagnostic criteria are present. Diagnosis of Legius syndrome has a different psychological impact compared to NF1, and permits to avoid the psychological stress due to possible complications of NF1, such as optic pathway glioma, learning difficulties, social and emotional difficulties, skeletal problems, development of neurofibromas [15]. Since the principal complications associated with Legius syndrome concern the neuro-behavioral field, once the diagnosis is certain, it is recommended to begin a follow-up program in order to identify any problems and intervene as soon as possible. This will make possible to reassure parents that neuro-behavioral development in patients with Legius syndrome presents less severe problems than in patients with NF1 [8, 16, 17].

We cannot consider PP as a feature of Legius syndrome, but the reported association between some RASopathies and CPP supports a link between these conditions, probably consequent to the involvement of RAS-MAPK pathway. Past research has in fact described cases of CPP in patients with RASophaties, in particular with cardio-facio-cutaneous syndrome (having BRAF mutation) [18] and with epidermal nevus syndrome (having HRAS mutation) [19, 20]. More recently, clinical studies have reported the cases of four patients, two with Costello syndrome and two with cardio-facio-cutaneous syndrome, who also presented CPP. In all of them, brain MRI was performed and no abnormalities were found. Nobody had hydrocephalus or ventriculomegaly, typical characteristics of these syndromes, causing CPP [14, 21]. Consequently, it is possible to hypothesize a role of the RAS-MAPK pathway in the genesis of CPP. It is in fact known that the RAS-MAPK pathway is involved in the regulation of the GnRH receptor signaling cascades. GnRH receptor signaling results in secretion of LH and FSH from the pituitary gland and stimulation of sex steroid production by the gonads. Therefore, genetic abnormalities in this pathway could theoretically lead to alterations during puberty, anyhow the exact mechanism at the basis of the precocious activation of hypothalamic-pineal-gonadal axis remains unclear [14, 21]. It is also possible that, in children with developmental delay, PP might occur as an indirect result of the gene mutation and might derive from disease-associated hypothalamic dysfunction, which could occur even without causative structural brain abnormalities. Nevertheless, our patient is not classified as affected by developmental delay but she only presents a mild disorder in attention maintenance. Therefore, according to previous literature we suggest a detailed investigation of the timing of puberty among patients with RASopathies [18, 22, 23] and a special care for girls with Legius syndrome, in order to identify possible signs of PP.

In conclusion, we report for the first time a case of CPP in a girl with Legius syndrome. In presence of CPP in children with characteristic café-au-lait macules, we suggest to perform genetic analysis in order to reach a definitive diagnosis between NF1 and Legius syndrome. Finally, further studies on timing of puberty in patients with RASopathies are needed to better elucidate if this clinical association is casual or secondary to their clinical condition.

## Abbreviations

NF1: Neurofibromatosis type1
PP: Precocious puberty
CPP: Central precocious puberty
PPP: Peripheral precocious puberty
MAS: McCune-Albright syndrome
SD: Standard deviation
MRI: Magnetic resonance imaging
NGS: Next generation sequencing
ACMG: American College of Medical Genetics
ADHD: Attention deficit hyperactivity disorder
